# *Staphylococcus aureus* Activates the NLRP3 Inflammasome in Human and Rat Conjunctival Goblet Cells

**DOI:** 10.1371/journal.pone.0074010

**Published:** 2013-09-10

**Authors:** Victoria E. McGilligan, Meredith S. Gregory-Ksander, Dayu Li, Jonathan E. Moore, Robin R. Hodges, Michael S. Gilmore, Tara C. B. Moore, Darlene A. Dartt

**Affiliations:** 1 Schepens Eye Research Institute/Massachusetts Eye and Ear and Department of Ophthalmology, Harvard Medical School, Boston, Massachusetts, United States of America; 2 Centre for Molecular Biosciences, University of Ulster, Coleraine, N. Ireland; 3 Cathedral Eye Clinic, Belfast, N. Ireland; 4 Departments of Ophthalmology and Microbiology and Immunobiology, Harvard Medical School, and Massachusetts Eye and Ear, Boston, Massachusetts, United States of America; UC Berkeley, United States of America

## Abstract

The conjunctiva is a moist mucosal membrane that is constantly exposed to an array of potential pathogens and triggers of inflammation. The NACHT, leucine rich repeat (LRR), and pyrin domain-containing protein 3 (NLRP3) is a Nod-like receptor that can sense pathogens or other triggers, and is highly expressed in wet mucosal membranes. NLRP3 is a member of the multi-protein complex termed the NLRP3 inflammasome that activates the caspase 1 pathway, inducing the secretion of biologically active IL-1β, a major initiator and promoter of inflammation. The purpose of this study was to: (1) determine whether NLRP3 is expressed in the conjunctiva and (2) determine whether goblet cells specifically contribute to innate mediated inflammation via secretion of IL-1β. We report that the receptors known to be involved in the priming and activation of the NLRP3 inflammasome, the purinergic receptors P2X4 and P2X7 and the bacterial Toll-like receptor 2 are present and functional in conjunctival goblet cells. Toxin-containing *Staphylococcus aureus (S. aureus)*, which activates the NLRP3 inflammasome, increased the expression of the inflammasome proteins NLRP3, ASC and pro- and mature caspase 1 in conjunctival goblet cells. The biologically active form of IL-1β was detected in goblet cell culture supernatants in response to *S. aureus,* which was reduced when the cells were treated with the caspase 1 inhibitor Z-YVAD. We conclude that the NLRP3 inflammasome components are present in conjunctival goblet cells. The NRLP3 inflammasome appears to be activated in conjunctival goblet cells by toxin-containing *S. aureus* via the caspase 1 pathway to secrete mature IL1-β. Thus goblet cells contribute to the innate immune response in the conjunctiva by activation of the NLRP3 inflammasome.

## Introduction

NOD Like Receptors (NLRs) have in recent years been evidenced to be guardians of the body. They are intracellular microbial and non-microbial sensors and include three main proteins; NLRP1, NLRP3 and NLRC4. [1] Each of which are known to form inflamamasomes, differing mainly in their activation stimuli. The assembly and activation of an elaborate multi-protein complex: nucleotide-binding oligomerization domain (NACHT), leucine rich repeat (LRR) domain, and pyrin domain-containing protein 3 (NLRP3) inflammasome is responsible for the recruitment of caspase 1 that processes IL-1β into a mature and biologically active form. [1] The NLRP3 inflammasome consists of the NLRP3 protein, which senses intracellular triggers resulting in oligomerization [2] that subsequently interacts with ASC (apoptosis-associated speck-like protein) through homotypic interactions of the pyrin domain. [1] ASC then interacts with pro caspase 1 resulting in cleavage and activation of caspase 1, which in turn cleaves pro IL-1β to its active form. [1], [3], [4].

NLRP3 is activated by a plethora of stimuli such as endogenous molecules including urate crystals, adenosine trisphosphate (ATP), and particulate matter such as silica and asbestos. [5]–[7] Bacterial stimuli such as Staphylococcus aureus (*S. aureus),* bacterial pore-forming toxins, bacterial RNA, and bacterial cell wall components lipopolysaccharide (LPS), lipoteichoic acid (LTA) and muramyl dipeptide (MDP) also activate NLRP3. [6], [8]–[10] The mechanism of activation is not yet fully understood, but the processing of IL-1β via the inflammasome has been demonstrated to involve two pathways. [11] First, transcription of the pro form of IL-1β is initiated by activation of the Toll-like receptor (TLR) induced NFκB pathway. IL-1β is then cleaved to produce the biologically active and secreted form by the activation of the caspase 1 pathway via inflammasome activation. The purinergic receptors P2X4 and P2X7 have been shown to be involved in inflammasome activation, initiated by Danger Associated Molecular Patterns (DAMPs) such as ATP. [6], [12]. Purinergic receptor stimulation leads to increased intracellular [Ca^2+^]_i_, potassium efflux from the cell, and subsequent generation of reactive oxygen species [13]–[15].

To date most of the work in this area has utilized immune cells such as macrophages as the working model of the NRLP3 inflammasome, demonstrating the classic model of priming, followed by a second specific stimulus. The two stimuli usually activate a TLR and a purinergic receptor of the P2X family, usually P2X4 or P2X7 respectively. It remains unknown whether this model is also true in other cell types. Interestingly, NLRP3 is reported to be highly expressed in wet mucosal epithelial membranes including the oropharynx, esophagus, ectocervix, and urothelial layer of the bladder, but is not expressed in the stomach, intestine, or lung. [16] However, research on the activation and function of NLRP3 in these wet mucosal epithelia is limited.

The wet mucosal membrane of the eye, the conjunctiva, is exposed to the environment and at the same time highly sensitive to the damaging effects of inflammation. The ocular surface therefore requires a carefully balanced mechanism to initiate inflammation only when absolutely necessary. While inflammation is sometimes necessary to clear an invading pathogen, damaging (chronic) inflammation is both the cause and consequence of most ocular surface conditions including bacterial infections, Stevens-Johnson syndrome, giant papillary conjunctivitis, seasonal allergic conjunctivitis, neurotrophic keratitis, ocular mucous membrane pemphigoid, and alkali and thermal burns. [17] Therefore, a need exists to understand the pathways that regulate inflammation, to provide a basis for the development of new, more targeted therapies aimed at preventing the non-specific tissue damage resulting from chronic inflammation.

Conjunctival goblet cells have been shown to be important in defense against ocular surface infections via mucin secretion. [18], [19] In previous studies we found atypical bacteria on the ocular surface of patients with overt inflammation [20] and demonstrated that increased bacterial flora was associated with reduced conjunctival goblet cell density, a marker of dry eye syndrome and ocular surface inflammation. [17] We also found altered levels of goblet cell mucin secretion in severe dry eye patients. [21] It was therefore of interest to investigate the role of goblet cells in regulating inflammation that can be induced by bacteria.

The literature to date is limited in regard to the function of the NLRP3 inflammasome in non-immune cells and to the best of our knowledge, no published report exists on the expression and function of inflammasomes in conjunctival epithelial cells. The objectives of the present study were to: (1) determine whether NLRP3 is expressed in the conjunctiva and (2) determine whether goblet cells specifically contribute to innate mediated inflammation via secretion of IL-1β. In the studies presented herein, we report the constitutive expression of pro IL1-β and the constitutive presence of all components of the NLRP3 inflammasome in the conjunctival goblet cells. We also report the activation of the NLRP3 inflammasome appears to be stimulated in conjunctival goblet cell cultures by stimulation with the gram positive bacterium *S. aureus,* which is commonly associated with ocular surface infections [23], [24] and is a specific activator of the NLRP3 inflammasome [15], [17]–[19].

## Materials and Methods

### Animals

Male Sprague Dawley rats weighing between 125 g and 150 g were obtained from Taconic Farms (Germantown, NY). Rats were anesthetized with CO_2_ for 1 min, decapitated, and the bulbar and fornical conjunctiva removed from both eyes. All experiments were carried out in accordance the Guide for the Care and Use of Laboratory Animals of the National Institutes of Health and were approved by the Schepens Eye Research Institute Animal Care and Use Committee.

### Human Material

The human eyes used for immunohistochemistry were obtained from the San Diego Eyebank (San Diego, CA; www.sdeb.org). Human conjunctival tissue was obtained from patients during ocular surgery. The tissue, which would normally be discarded, was obtained with written informed consent from each donor using a protocol that adhered to the tenets of the Declaration of Helsinki, and approved by the Schepens Eye Research Institute and Massachusetts Eye and Ear Human Studies Internal Review Boards. Three patients (two male and 1 female age range, 58–73) underwent surgury for pars plana vitrectomy, pars plana vitrectomy with membrane peeling, and scleral buckle. Conjunctival tissue was also obtained from Heartland Lions Eye Bank (Kansas City, MO; www.hleb.org).

### Cell Culture

Goblet cells from rat and human conjunctiva were grown in organ culture as described previously. [22]–[25] Pieces of minced tissue were placed in RPMI 1640 medium supplemented with 10% fetal bovine serum (FBS), 2 mM glutamine (Lonza, Walkersville, MD), and 100 mg/ml penicillin-streptomycin. The tissue plug was removed after nodules of cells were observed. As described previously [22]–[25], cells were identified as goblet cells by the following characteristics: 1) morphology by light microscopy both bright-field and following histochemical staining with periodic acid–Schiff’s reagent (indicates secretory product); 2) positive staining with the lectins *Ulex europaeus* agglutinin type I (UEA-I), specific for rat, or *Helix pomatia* agglutinin (HPA) lectin, specific for human; and antibodies to MUC5AC (all three stain secretory product) and cytokeratin 7 (detects cell body); and 3) negative staining with antibody to cytokeratin 4.

### Immunohistochemistry

For immunofluorescence microscopy of intact conjunctiva, eyes were enucleated with the lids intact and fixed in 4% formaldehyde in phosphate buffered saline (PBS, 145 mM NaCl, 7.3 mM Na_2_HPO_4_, and 2.7 mM NaH_2_PO_4_ (pH 7.2) overnight at 4°C. Eyes were embedded in paraffin. Sections (6 µm) were placed on slides and kept at −20°C until use. For immunohistochemistry, human sections were deparaffinized and antigen retrieval was used prior to staining for NLRP3. For immunohistochemistry of cultured cells, primary cells were grown on glass coverslips and then fixed in methanol before use for cytokeratin analysis and fixed in 4% paraformaldehyde for all other protein analyses. The lectin UEA-I conjugated to fluorescein isothiocyanate (FITC) (Sigma-Aldrich, St. Louis, MO) was used at a dilution of 1∶500; and the lectin HPA conjugated to FITC (Pierce, Rockford, IL) was used at a dilution of 1∶1000. UEA-I binds to the goblet cell secretory product carbohydrate α-L fucose on terminal sugars present on mucins stored in secretory granules of goblet cells. HPA similarly identifies L-galactosamine on terminal sugars of mucins. Antibodies used were mouse anti-human NLRP3 (1∶50, Enzo Life Sciences, catalog number 804–819-C100) and rabbit anti-rat caspase 1 (1∶20) (Enzo Life Sciences, Plymouth, PA); rabbit anti-rat ASC (1∶50) (Santa Cruz Biotechnology, CA, USA); anti-rabbit P2X4 and P2X7 (1∶50) (Alomone Labs, Jerusalem, Israel); anti-rabbit TLR2 (1∶100) (Santa Cruz Biotechnology, CA). DAPI was added to the mounting medium to identify cell nuclei. Secondary antibodies were conjugated to Cy3 (Jackson ImmunoResearch Laboratories, West Grove, PA) and used at a dilution of 1∶300. Negative controls included use of the isotype controls for rabbit (Santa Cruz Biotechnology) and mouse (Millipore, MA, USA) antibodies.

### Measurement of [Ca^2+^]_i_

Cultured goblet cells were seeded onto glass-bottom 35 mm petri dishes (MatTek, Ashland, MA) and allowed to attach overnight at 37°C. Cells were then incubated with 8 µM fura 2-AM (Invitrogen, Carlsbad, CA) for 1 h at 37°C, in buffer (119 mM NaCl, 4.8 mM KCl, 1.0 mM CaCl_2_, 1.2 mM MgCl_2_, 1.2 mM KH_2_PO_4_, and 25 mM NaHCO_3_ supplemented with 10 mM HEPES, 5.5 mM glucose, 250 mM sulfinpyrazone, and 0.5% BSA) and then stimulated with ATP (0.1 µM–5 mM). In separate experiments cells were pre-incubated with LTA (1 µg/ml or 10 µg/ml) for 5 h, loaded with fura-2 for 1 h, and then stimulated with ATP (5 mM). Fluorescent images of cells were recorded and analyzed with a digital fluorescence imaging system (InCyte Im2, Intracellular Imaging, Cincinnati, OH). Peak [Ca^2+^]_i_ was calculated by subtracting the basal values (before the addition of agonist) from the peak calcium value.

### Western Blotting Experiments

Pieces of rat conjunctiva or goblet cells cultured in 6 or 12 well plates were lysed in RIPA buffer. The lysate was centrifuged at 10,000×g for 10 min at 4°C. Sample buffer (4X) was added to the lysate, and proteins separated by SDS-PAGE through 10% polyacrylamide, and transferred to nitrocellulose membrane to be processed for Western blot. RIPA used for Western blotting contained 10 mM Tris-HCl (pH 7.4), 150 mM NaCl, 1% deoxycholic acid, 1% Triton X-100, 0.1% SDS, and 1 mM EDTA. THP-1 cell lysate (Santa Cruz Biotechnology, Santa Cruz, CA) was used as the positive control for detection of caspase-1, ASC, and Pro-IL-1β [1], [28]–[31]. Both untransfected HEK293 and HEK293 cells transfected with NRLP3 were from Imgenex (San Diego, CA).

The primary antibodies used for Western blots with the following dilutions: 1∶200 for NLRP3 (R&D Systems, Minneapolis, MN), 1∶50 for ASC, and 1∶1000 for caspase 1, (R&D Systems, Minneapolis, MN) 1∶100 for P2X4 and P2X7, 1∶200 for TLR2 and 1∶1000 for IL-1β (R&D Systems, Minneapolis, MN). Anti-mouse secondary antibody conjugated to HRP was from Santa Cruz (Santa Cruz, CA) and was used at a dilution of 1∶2000. Alternatively, anti-rabbit secondary antibody conjugated to HRP (Millipore, Billerica, MA) was used at a dilution of 1∶5000, anti-sheep secondary antibody was from R&D Systems and used at a dilution of 1∶2500, and anti-goat secondary antibody (Santa Cruz Biotechnology, Santa Cruz, CA) and was used at 1∶5000. Immunoreactive bands were visualized by the Enhanced Chemiluminescence method (Thermo Scientific, Rockford, IL).

### *S. aureus* (RN6390) Culture and Challenge of Rat Conjunctival Goblet Cells

*S. aureus* (RN6390) was cultured as previously described. [26] Briefly *S. aureus* was cultured at 37°C overnight with continuous agitation. This pre-culture was then diluted and grown at 37°C to an OD595 nm of 0.5 (early log phase). After centrifugation at 500×g for 10 min, the supernatant was discarded and bacteria resuspended in RPMI-1640 medium with 1% FBS. Rat conjunctival goblet cells were seeded in 12 well culture plates (500 cells per well) in medium without antibiotics 24 h prior to infection, and some cultures were preincubated with the caspase 1 inhibitor Z-YVAD (10 µM) for 30 min prior to infection with *S. aureus* (Biovision, California). *S. aureus* were added at a multiplicity of infection (MOI) of 20, 40 or 60, and incubated for 6 h at 37°C in 5% CO_2_. Cultures were then treated for an additional 2 h with 5 mM ATP or no additions. At 8 h cell culture supernatant was collected for analysis of IL-1β by ELISA (R&D Systems) that was performed according to the manufacturer’s instructions. The ELISA detected IL-1β over a range of 1 pg/ml –2500 pg/ml. In parallel, cells were lysed in RIPA buffer and protein collected for analysis by western blot. Cell viability assays using 0.05% (w/v) trypan blue (Sigma-Aldrich, St. Louis, MO) were performed in preliminary experiments to ascertain optimum time points to examine infection.

### FLICA Assay for Active Caspase 1 Analysis

Active caspase 1 was detected using a fluorescent inhibitor of caspases (FLICA, Immunochemistry Technologies, Bloomington, MN, USA), according to the manufacturer’s instructions. Cells were cultured, with or without *S. aureus* with or withoutATP as previously described and 10 µl of a 30×FLICA solution were added. The culture plates were covered with aluminium foil and incubated 1 h at 37°C in 5% CO_2_. Following incubation, the cells were washed with 2 ml of wash buffer, and then stained with Hoechst 33342 stain (0.5% v/v) according to manufacturer’s instructions. Stained cells were viewed on an inverted phase contrast microscope equipped for epifluorescence (Eclipse TE 300, Nikon, Tokyo, Japan; UV filter with excitation 490 nm, emission >520 nm for green fluorescence of caspase 1 positive cells, and excitation 365 nm, emission 480 nm for visualization of blue fluorescence from nuclear staining by Hoechst stain). The total number of nuclei in four (40X) fields of view was counted, and the number of cells staining green (indicative of active caspase 1) was expressed as a percentage of the total.

### Statistical Analysis

All results are representative of three independent experiments. Western blot results were expressed as the fold-increase above basal. Results are presented as mean±SEM. Data were analyzed by Student *t* test. A *p* value<0.05 was considered statistically significant.

## Results

### The NLRP3 Inflammasome Components NLRP3, ASC and Caspase 1 are Constitutively Present in the Human and Rat Conjunctiva

**Figure 1:**
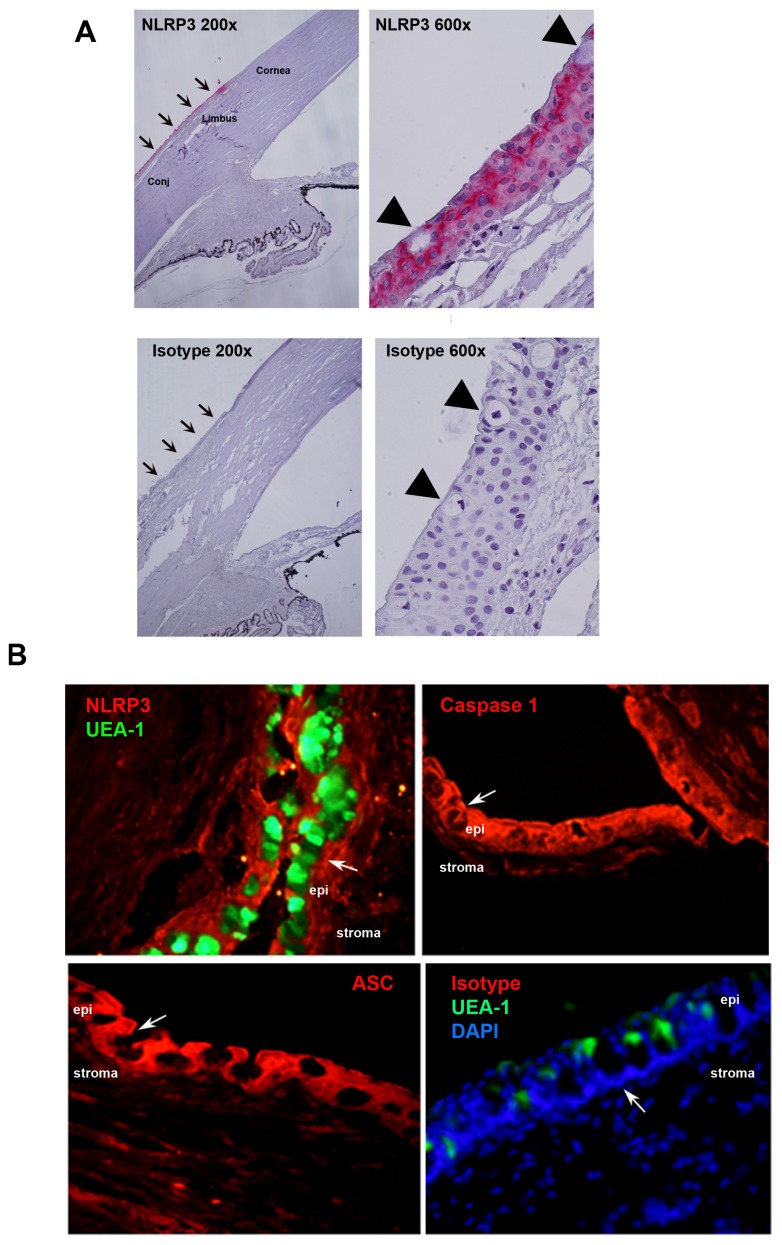
Human conjunctiva and rat conjunctiva constitutively express the NLRP3 inflammasome components. Human conjunctiva was analyzed by immunohistochemistry and NLRP3 was shown to be highly expressed in the epithelium as indicated by pink staining (A). Arrows indicate epithelial layer of conjunctiva; arrowheads indicate goblet cells. Isotype controls were negative. Rat conjunctiva was analyzed by immunofluorescence microscopy (B) and all three of the inflammasome components NLRP3, caspase 1, and ASC were identified as demonstrated by the red peri-nuclear staining. The green UEA staining indicates goblet cell secretory product, denoting the location of goblet cells in the conjunctiva. Arrows indicate goblet cells. The mouse isotype controls (B) were negative as were the rabbit isotype controls (not shown). Epi, epithelium.

Unlike NLRP1 that is located in the nuclei, NLRP3 is usually found in the cytoplasm. [16] Immunohistochemical analysis identified the NLRP3 protein in the human conjunctiva (Figure 1A). The lower power micrographs demonstrate the NLRP3 immunoreactivity is present in the conjunctiva but terminates at the end of the limbus adjacent to the peripheral cornea. NLRP3 was identified in the cytoplasm of both goblet and stratified squamous cells. The higher power micrographs indicate that NRLP3 immunoreactivity is present throughout the cell layers of the conjunctival epithelium and in both goblet and stratified squamous cells. The isotype negative control had no visible staining.

In rat conjunctival sections goblet cells were identified by UEA-1 binding that indicates the position of the secretory granules with the goblet cell body subjacent to this staining (Figure 1B, first panel on left). In the absence of UEA-1, goblet cell secretory granules and hence goblet cell bodies can be identified by the large black holes in the epithelium (Figure 1B second through fourth panels from left). The presence of NLRP3 inflammasome components NLRP3, ASC and caspase 1 were investigated in rat conjunctival sections. All three inflammasome components were identified in the cytoplasm of both goblet and stratified squamous cells (Figure 1B). The inflammasome components were strongly expressed throughout the epithelium of the conjunctiva, but were not detected in the stroma. NRLP3 was found diffusely throughout the cytoplasm of goblet and stratified squamous cells.

### The NLRP3 Inflammasome Components (NLRP3, ASC, Caspase 1) and pro IL-1β are Constitutively Present in Human and Rat Conjunctival Goblet Cells

**Figure 2:**
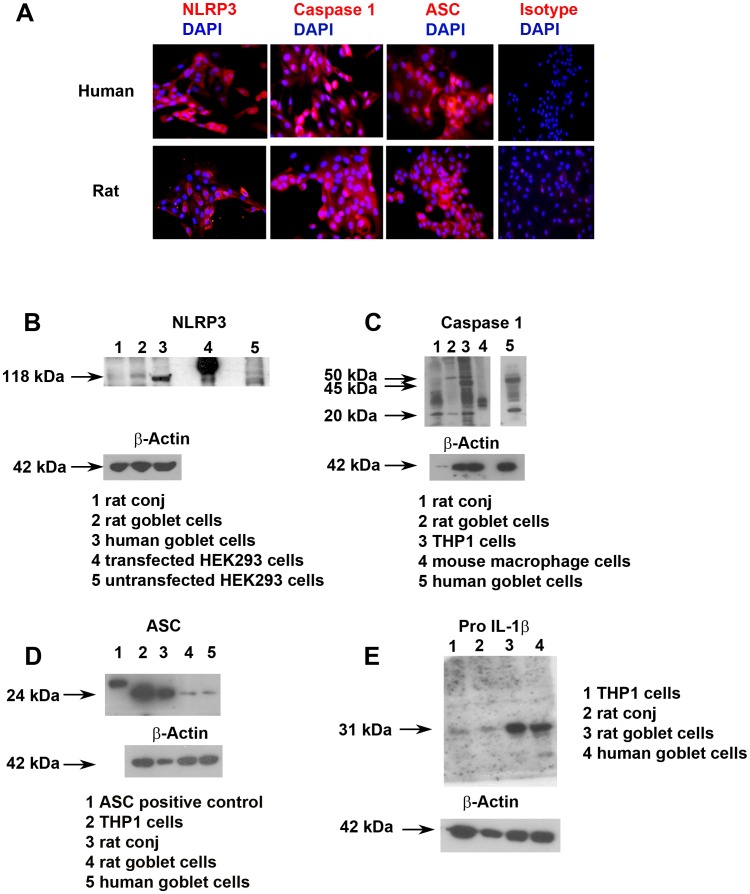
Constitutive expression of the NLRP3 inflammasome components in human and rat conjunctival goblet cells. Primary cultures of human or rat goblet cells were analyzed by immunofluorescence microscopy after staining with antibodies against inflammasome components NLRP3, caspase 1, and ASC (**A**). All three components were identified as indicated by the red peri-nuclear staining pattern. Rabbit isotype controls (shown) and mouse isotype controls (not shown) were negative. The presence of NLRP3 (**B**), caspase 1 (**C**), ASC (**D**) and IL-1β (**E**) were confirmed by western blot analysis in lysates from human goblet cells and rat conjunctiva and goblet cells. Please note that the positive controls for NLRP3 and ASC are transfected cells lines overexpressing the protein and thus the amount of protein loaded for the western blots was below the detection level of the β-actin antibody. In addition, the NLRP3 is tagged with His tag while the ASC is tagged with a FLAG tag causing these proteins to run at a higher molecular weight than the native molecules.

Primary cultures of human and rat conjunctival goblet cells were characterized using the goblet cell specific markers MUC5AC, HPA and cytokeratin 7 as described previously. [23], [24], [27] These cultures were negative for the stratified squamous epithelial cell marker cytokeratin 4 (data not shown). All NLRP3 inflammasome components NLRP3, ASC and caspase 1 were detected by immunofluorescence microscopy in both human and rat goblet cell cultures (Figure 2A), with each demonstrating a cytoplasmic staining pattern. The isotype negative control had no visible staining.

Western blotting analysis was performed on homogenates prepared from rat conjunctival tissue, cultured rat and human conjunctival goblet cells, transfected HEK293 cells as the positive control and untransfected HEK293 cells as the negative control. NRLP3 protein was present as a 118 kDa protein in all the conjunctival samples and the positive control, but not in the negative control (Figure 2B). Please note that the positive control for NLRP3 is a transfected cell line overexpressing NLRP3 flagged with a His tag which runs at a higher molecular weight than native NLRP3.

Caspase 1 consists of several isoforms. Caspase 1α is a 50 kDa protein while caspase 1β, δ and γ have molecular weights from 30–45 kDa. Active capase 1 is a 20 kDa protein. In rat conjunctiva, caspase 1 was present as bands at 50, 45, and 20 kDa (Figure 2C) indicating the presence of several isoforms of caspase 1 as well as active caspase 1. In rat goblet cells caspase 1 was seen at 50 and 20 kDa indicating the presence of caspase 1α and active caspase 1. In the positive control THP1 cells, caspase 1 was detected as bands at the 50, 45, and 20 kDa level indicating the presence of caspase 1 and several additional isoforms along with active caspase 1 [1], [28]. In human goblet cells caspase 1 was detected as a large band at 45 kDa and a band at 20 kDa indicating the presence of one or more isoforms of caspase 1.

ASC is a 24 kDa protein. A band at this molecular weight was detected in THP-1 cells as well as rat conjunctiva, rat and human goblet cells (Figure 2D) [29]. An additional positive control used for ASC was ASC protein tagged with FLAG that ran at a higher molecular weight (30 kDa) than native ASC. This control protein was detected at the expected molecular weight.

Pro IL-1β exists as a 31 kDa protein that is cleaved by caspase 1 to produce mature or active IL1β that has a molecular weight of 17 kDa and is secreted. A band at 31 kDa was detected in the positive control THP-1 cells, rat conjunctiva and goblet cells, and human goblet cells (Figure 2E) [30], [31]. No band was detected at 17 kDa in any of the samples.

### The Purinergic Receptors P2X4 and P2X7 and the Bacterial Receptor TLR2 are Present in Rat Conjunctival Goblet Cells

**Figure 3:**
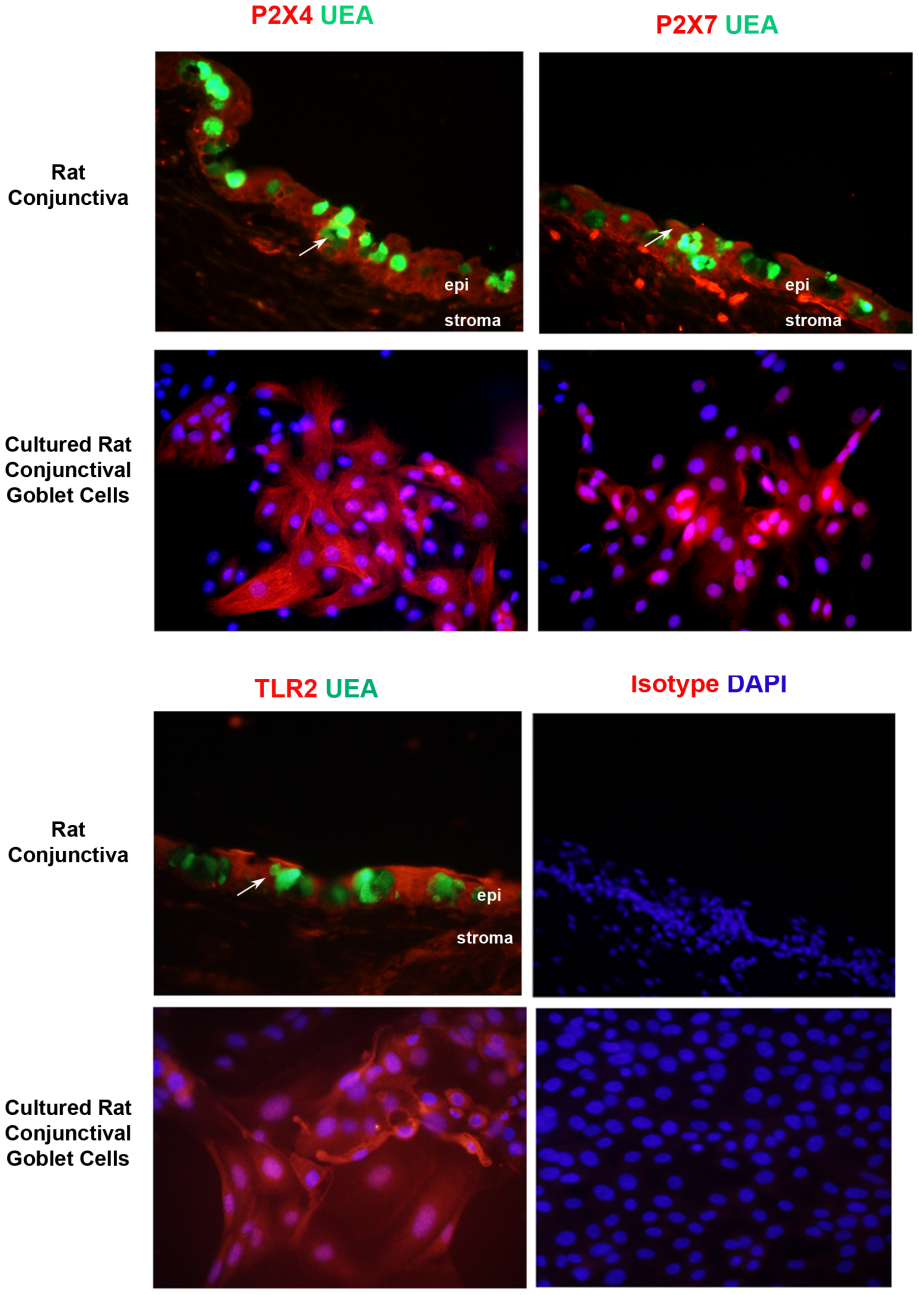
Purinergic receptors P2X4, P2X7, and TLR2 are expressed in the rat conjunctiva and rat goblet cell cultures. All three receptors were identified by red immunofluorescent staining. UEA stains goblet cell secretory products green, allowing the identification of goblet cells within the conjunctiva. Rabbit isotype controls were negative. Arrows indicate location of goblet cells.

The ionotropic purinergic receptors P2X4 and P2X7 are involved in inflammasome activation via binding of extracellular ATP, [12], [13] and TLR2 is critical in the priming of the inflammasome and activated by lipotechoic acid (LTA) a component of the gram positive *S. aureus* cell wall [32] or by *S. aureu*s binding. [10] All three receptors are present in both goblet and stratified squamous cells of the rat conjunctiva with limited expression in the stroma (Figure 3top). In the conjunctiva P2X4 is located on the membranes surrounding the goblet and stratified squamous cells. In cultured goblet cells P2X4 immunoreactivity is detected on the plasma membranes and contractile stress fibers located within the cytoplasm. P2X7 immunoreactivty in the conjunctiva demonstrated intense staining in the cell bodies of the goblet cells with diffuse staining in the cytoplasm of the stratified squamous cells (Figure 3 top). In cultured goblet cells the P2X7 positivity was located diffusely in the cytoplasm. TLR2 was localized in goblet cell bodies and in stratified squamous cells (Figure 3 bottom). TLR2 immunoreactivity was present in the cytoplasm and plasma membranes in cultured goblet cells.

**Figure 4:**
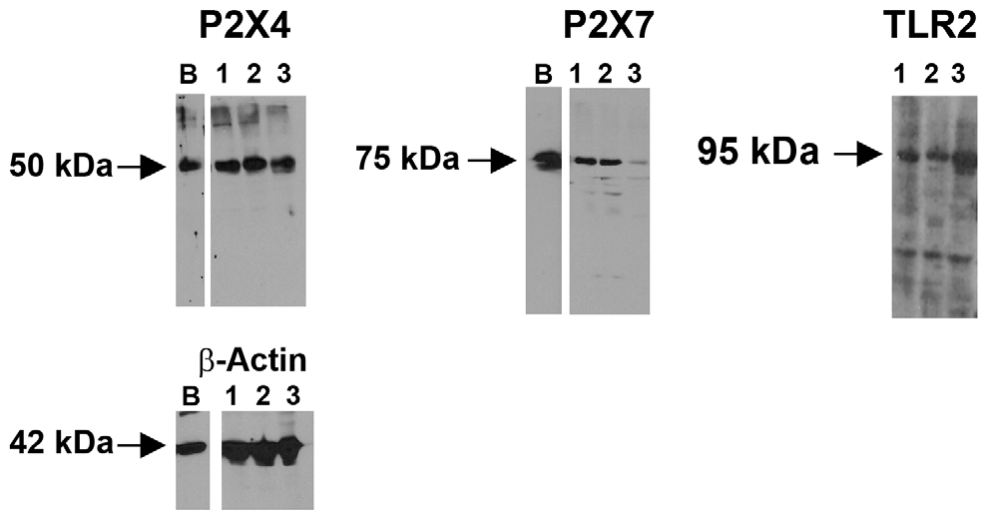
Purinergic receptors P2X4, P2X7 and TLR2 are expressed in rat goblet cell cultures. All three receptors were identified by western blot. Lanes 1–3 represent separate animals. B is the positive control of rat brain. The β-actin blot is for both P2X4 and P2X7 receptors blots.

The presence of P2X4, P2X7, and TLR2 was determined by western blotting analysis in rat conjunctival goblet cell homogenate from three different animals (Figure 4). Rat brain homogenate was used as a positive control. P2X4 was detected at its expected 50 kDa protein and the same molecular weight as the positive control of rat brain, P2X7 at 75 kDa and the same molecular weight as the positive control of rat brain, and TLR2 at 95 kDa. We also identified TLR 1, 4 and 6, and cluster of differentiation 14 (CD14) on rat goblet cells (data not shown).

**Figure 5:**
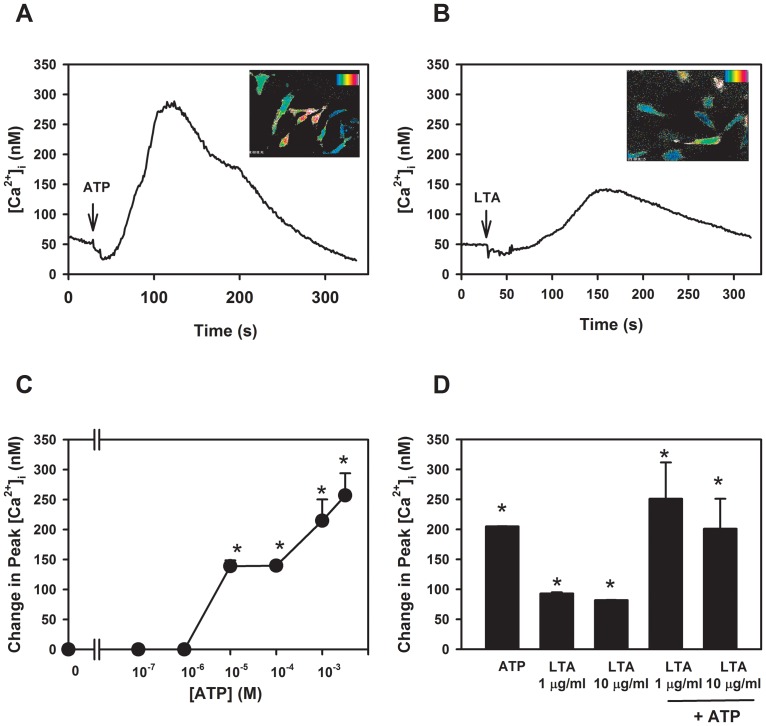
The purinergic receptors P2X4, P2X7, and TLR2 are functional in cultured rat goblet cells. Rat goblet cells were loaded with fura-2 and then stimulated with ATP and intracellular calcium response measured. A typical trace from one experiment with ATP (5 mM, **A**) or lipoteichoic acid (LTA, 10 µg/ml, **B**) is representative of 3 animals. Representative photographs of a single field of cells after treatment with ATP or LTA, with warmer colors indicating intracellular Ca^2+^ increase, are shown as insets. Peak [Ca^2+^]_i_ calculated for ATP (0.1 µM–5 mM) from 3 experiments, is shown in **C**. Peak [Ca^2+^]_i_ for goblet cells, also preincubated with LTA for 5 h and then loaded with fura-2 for 1 additional h before addition of ATP (5 mM), calculated from 3 experiments, is shown in **D**. Results are expressed as mean ± SEM. * indicates significance of *p*<0.05 from no addition (0).

Measurement of [Ca^2+^]_i_ was used to determine if the purinergic receptors (P2X4 and P2X7) were functional in rat goblet cells. Primary cultures of rat goblet cells were loaded with fura-2 and stimulated with ATP (10^−7^–5×10^−3^ M) a non-specific activator of P2X receptors. ATP increased [Ca^2+^]_i_ in the goblet cells with a rapid peak response that declined back to basal levels. ATP elevated [Ca^2+^]_i_ in a concentration dependent manner, with ATP from 10^−5^–5×10^−3^ M increasing [Ca^2+^]_i_ to significantly higher levels than in control (unstimulated) cells (Figure 5A and C). The highest peak increase over basal was 257.0±36.9 nM.

Rat goblet cells were also stimulated with LTA a potent activator of TLR2. [32] Stimulation with LTA (1 µg/ml or 10 µg/ml) resulted in intracellular Ca^2+^ responses that were significantly higher than control (unstimulated) goblet cells (Figures 5B and D). The average peak Ca^2+^ response of rat goblet cells to LTA (1 µg/ml) was 93 nM as compared to 204.7±0.3 nM for ATP (5×10^−3^ M) (Figure 5D). To mimic the two stimulus hypothesis of activation of the NRLP3 inflammasome, goblet cell cultures were pre-incubated with LTA (1 µg/ml or 10 µg/ml) for 5 h prior to loading with fura-2 for 1 additional hour, and then stimulated with ATP (5×10^−3^ M). Preincubation with LTA did not alter the intracellular Ca^2+^ response to ATP which was 251.0±60.6 nM and 201.0±50.0 nM for 1 µg/ml and 10 µg/ml respectively compared to ATP alone that was 204.7±0.3 nM (Figure 5D).

These experiments show that the purinergic receptors P2X4 and P2X7 and TLR2 are present and functional in rat goblet cells. They also demonstrate that bacterial LTA treatment does not alter the purinergic intracellular Ca^2+^ response.

### *S. aureus* Challenge with or without ATP Increases NLRP3, ASC, Caspase 1 and Pro IL-1β Protein Expression in Rat Conjunctival Goblet Cells

**Figure 6:**
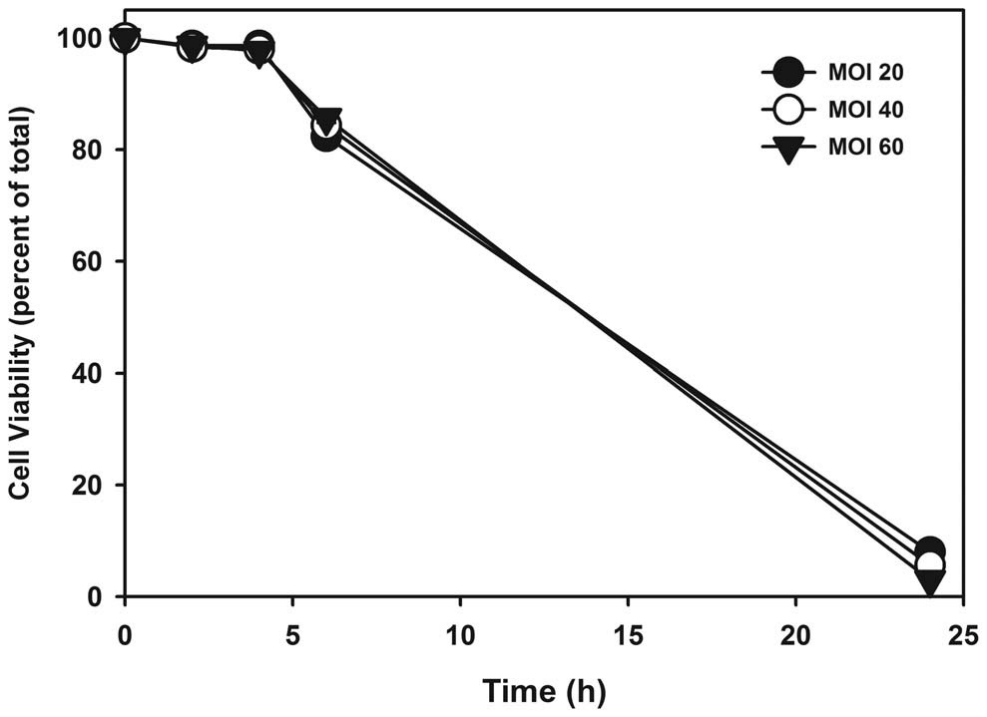
Effect of *S. aureus* on Goblet Cell Viability. Cultured rat goblet cells were incubated with *S. aureus* at MOIs of 20, 40, and 60 for 0–24 h, and cell viability was determined by trypan pan blue exclusion. Data is mean ± SEM from 3 independent experiments.

Rat goblet cell cultures were challenged with *S. aureus* (RN6390) at MOI 20, 40 or 60 over 24 h and cell viability determined using the trypan blue exclusion assay. These MOIs were chosen as they were shown to be effective in stimulating IL-1 β expression in human corneal epithelial cells in a previous study. [26] Over 80% of the cells remained viable at each MOI at 8 h before a rapid decline in cell viability (Figure 6). Therefore 8 h was chosen as the end point for subsequent experiments.

**Figure 7:**
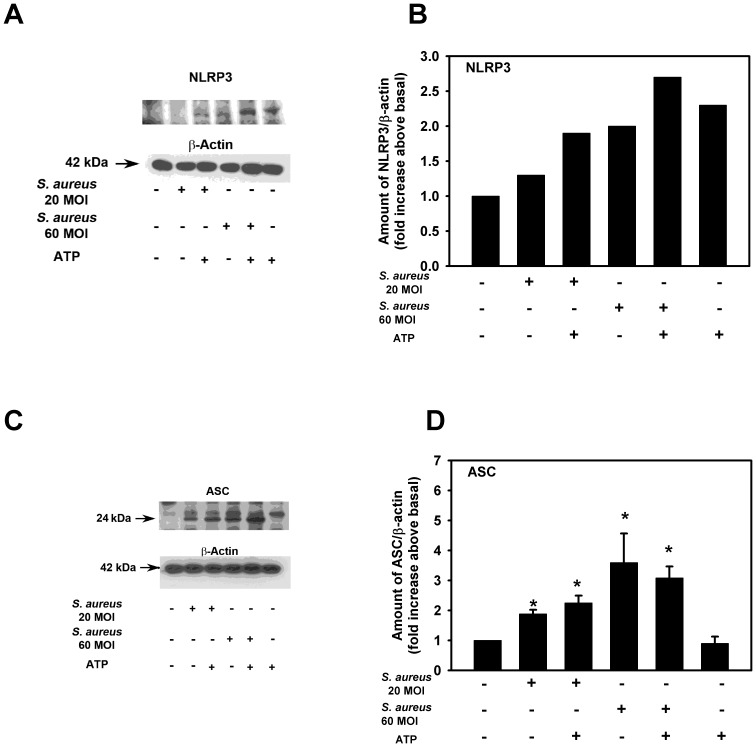
Effect of *S. aureus* on NLRP3 and ASC expression by cultured rat goblet cells. Cultured rat goblet cells were incubated with *S. aureus* (MOI 20 or 60) for 6 h. Cultures were treated for an additional 2 h with ATP (5 mM) or buffer alone. Cell lysates collected and analysed by western blot. Representative blots are shown in **A** and **C**. Lower, major band in ASC blot was scanned. Blots were scanned and quantified. Data from a single experiment, representative of two experiments, is shown in **B** and means ± SEM from 3 independent experiments are shown in **D**. * indicates significance of *p*<0.05 compared to no addition which was set to 1.

Cultured rat conjunctival goblet cells were incubated with *S. aureus* for 6 hr with or without ATP (5×10^−3^ M) for an additional 2 hr, cells homogenized and analysed for amount of NRLP3 and ASC. Incubation with *S. aureus* alone at 20 and 60 MOI increased the amount of NRLP3 over basal to 1.3 and 2.0 fold respectively (Figure 7A and B). Addition of ATP slightly increased the response to *S. aureus* and ATP added alone also increased the amount of NRLP3.

For ASC, *S. aureus* at 20 and 60 MOI significantly increased the amount of ASC compared to basal (Figure 7C and D). Addition of ATP did not further increase the amount of ASC at either concentration of bacteria. ATP alone did not alter the amount of ASC.

**Figure 8:**
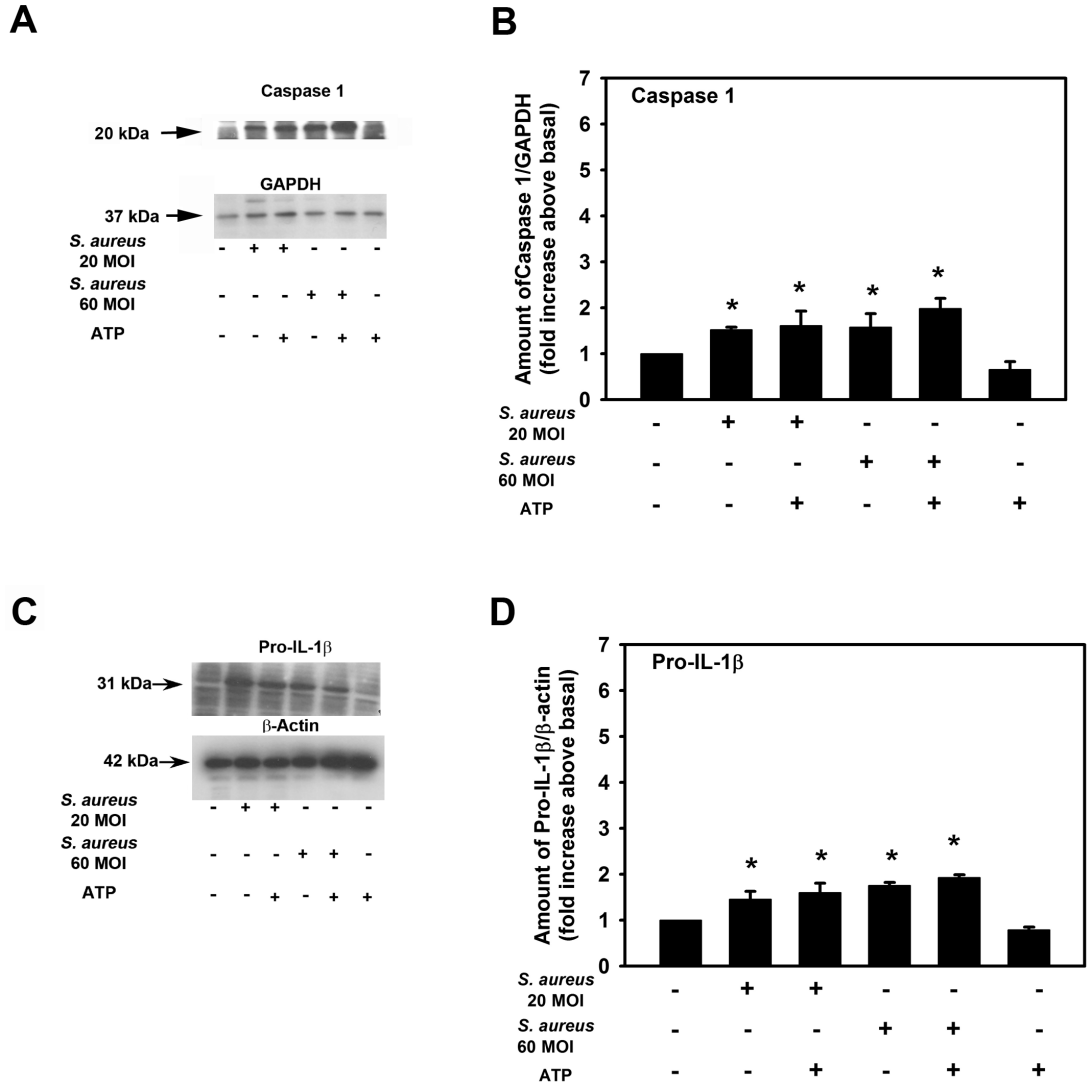
Effect of *S. aureus* on Caspase 1 and proIL-1β protein expression. Cultured rat goblet cells were incubated with *S. aureus* (MOI 20 or 60) for 6 h. Cultures were treated for an additional 2 h with ATP (5 mM) or buffer alone. Cell lysates were collected and analyzed by western blot. Representative blots are shown in **A** and **C**. Blots were scanned and mean ± SEM from 3 independent experiments are shown in **B** and **D**. * indicates significance of *p*<0.05 compared to no addition which was set to 1.

Results similar to those found with ASC were obtained for caspase 1 and pro IL-1β (Figure 8A, B, C and D). Goblet cells challenged with *S. aureus,* with or without ATP, also significantly increased caspase 1 and pro IL-1β protein expression as compared to basal levels. The level of caspase 1 and pro IL-1β protein expression was not significantly different between cells challenged only with *S. aureus* and *S. aureus* challenged with ATP.

Taken together these results suggest that *S. aureus* itself without addition of ATP can increase the amount of the constituents of the NRLP3 inflammasome including NLRP3, ASC, and caspase 1. In addition *S. aureus* increases the amount of pro IL-1β. ATP alone does not increase the level of ASC, caspase 1or pro IL-1β.

### *S. aureus* Challenge, with or without ATP, Leads to IL-1β Secretion from Rat Conjunctival Goblet Cells Dependent on Caspase 1 Activation

**Figure 9:**
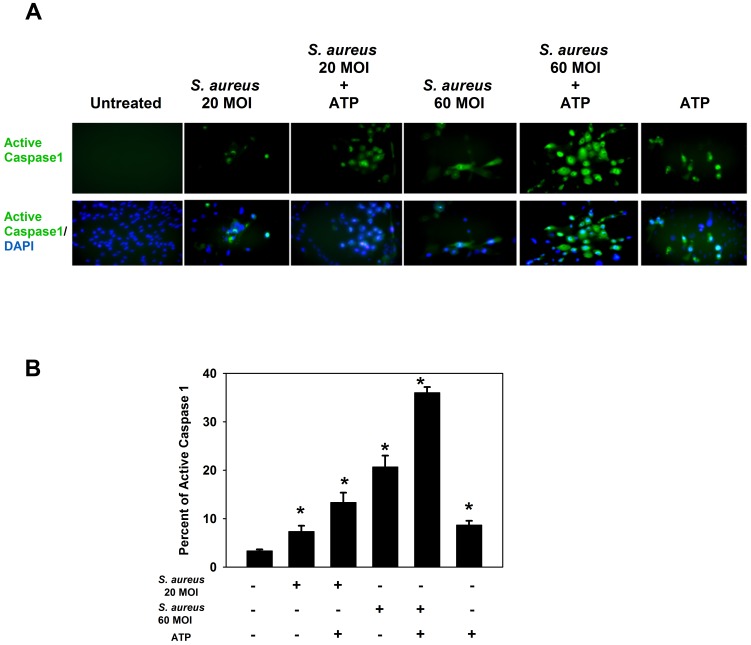
Active caspase 1 expression in rat goblet cells treated with *S. aureus* and ATP. Primary cultures of rat goblet cells were incubated with *S. aureus* (MOI 20 or 60) for 6 h. Cultures were treated for an additional 2 h with ATP (5 mM) or buffer alone. The FLICA reagent, which detects only active caspase 1, was added followed by the nuclear Hoescht stain and viewed by immunofluorescence microscopy. Representative micrographs are shown in **A**. The total number of nuclei in four fields of view and the number of cells with staining green (indicative of active caspase 1) were counted. Data is expressed as mean ± SEM from 3 independent experiments, and are shown in **B**. * indicates significance of *p*<0.05 compared to no addition, which was set to 1. Magnification 40×.

The active form of caspase 1 was detected in primary cultures of rat goblet cells challenged with *S. aureus,* with or without ATP (5×10^−3^ M), as determined by the FLICA assay (Figure 9A). *S. aureus* challenge alone, ATP treatment alone, or the combination of *S. aureus* with ATP treatment, resulted in significant activation of caspase 1 as compared to untreated cells. The greatest amount of caspase 1 activation was observed in cultures that were treated with the highest MOI of *S. aureus* (MOI 60 (21%)), and this activation was enhanced when ATP was added to the cultures (36%) (Figure 9B). It is important to note that there was no significant difference in cell viability between MOI 20 and MOI 60 (Figure 6).

**Figure 10:**
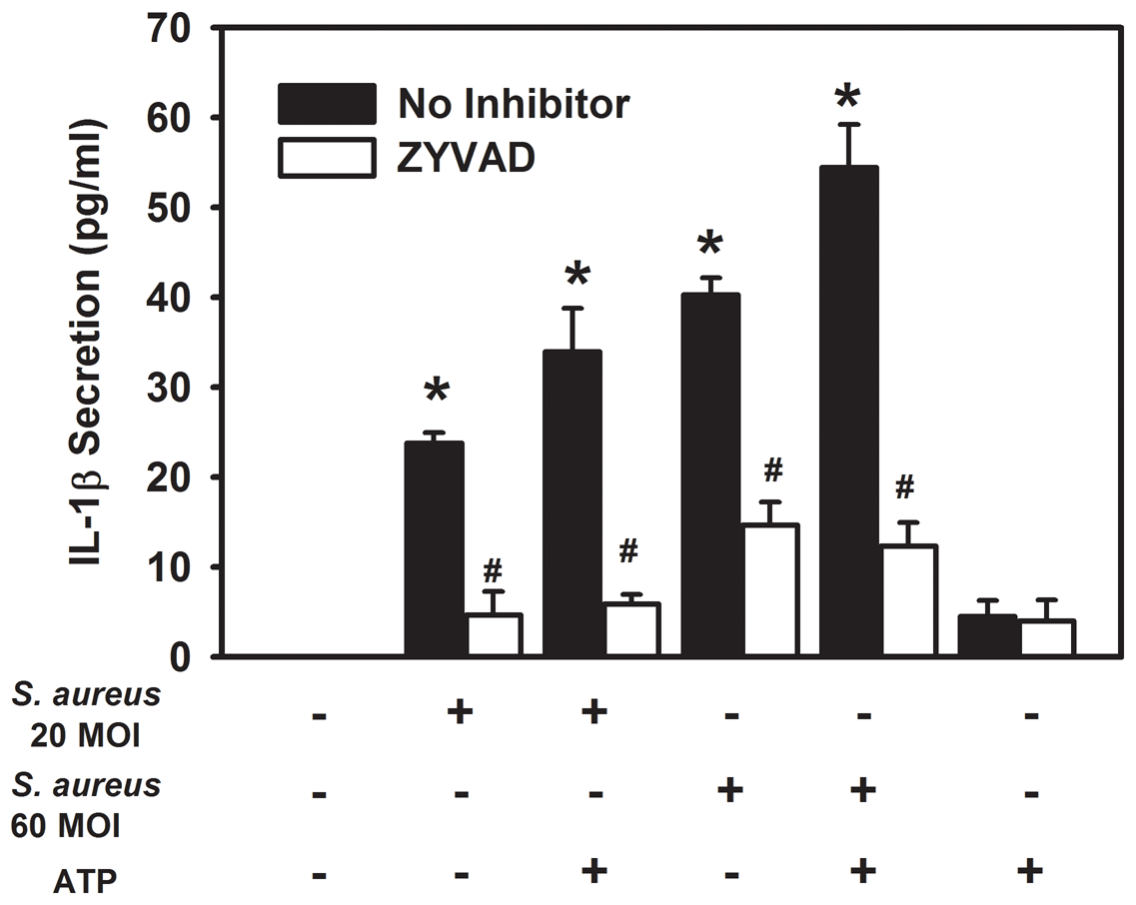
Effect of Inhibition of Caspase 1 on IL-1β secretion in response to *S. aureus* and ATP. Primary cultures of rat goblet cells were treated with or without the caspase 1 inhibitor Z-YVAD for 1 h and then incubated with *S. aureus* (MOI 20 or 60) for 6 h. Cultures were treated for an additional 2 h with ATP (5 mM) or buffer alone. Culture supernatant was removed and analyzed for IL-1β by ELISA. Data is expressed as mean ± SEM from 3 independent experiments. * indicates significance of *p*<0.05 compared to no addition. # indicates significance of *p*<0.05 compared to no inhibitor.

IL-1β secretion was measured by ELISA in the supernatant isolated from cultured rat conjunctival goblet cells stimulated by *S. aureus* at 20 and 60 MOI with or without ATP (5×10^−3^ M) or ATP alone. Challenge of rat goblet cells with *S. aureus,* with or without ATP, resulted in a significant increase in IL-1β secretion as compared to untreated cells (Figure 10). Cells challenged with *S. aureus* at an MOI 60 resulted in significantly more IL-1β secretion than from cells challenged with *S. aureus* at MOI 20 (40 pg/ml and 24 pg/ml respectively). IL-1β secretion from goblet cells was slightly increased when ATP was added to *S. aureus* stimulated goblet cell cultures, compared to *S. aureus* alone (34 pg/ml for *S. aureus* MOI 20 plus ATP compared to 24 pg/ml for *S. aureus* MOI 20 alone; and 55 pg/ml for *S. aureus* MOI 60 plus ATP compared to 40 pg/ml for *S. aureus* MOI 60 alone). Interestingly, IL-1β secretion from goblet cells treated with ATP alone was not significantly different from untreated cells (Figure 10), even in the presence of significantly elevated caspase 1 activity (Figure 9). Taken together, these data demonstrate the importance of a priming agent for the induction of pro IL-1β. In the absence of *S. aureus*, goblet cells treated with ATP alone fail to upregulate pro IL-1β. Therefore, even with the activation of caspase 1, there is a limited store of pro IL-1β to convert to mature IL-1β in the absence of a priming agent such as *S. aureus*.

The role of caspase 1 was confirmed using the caspase 1 inhibitor Z-YVAD. The addition of Z-YVAD to the rat goblet cell cultures challenged with *S. aureus,* with or without ATP, resulted in a significant decrease in IL-1β secretion with IL-1β secretion dropping from 54 pg/ml (no inhibitor present) to 12 pg/ml (inhibitor present) for cultures incubated with *S. aureus* MOI 60 and ATP (Figure 10). No significant difference was noted in IL-1β secretion between cells treated with ATP alone, and cells treated with ATP and the caspase 1 inhibitor Z-YVAD. These results provide evidence that *S. aureus* activates the secretion of IL-1β via the caspase 1 pathway.

## Discussion

We found that the NLRP3 inflammasome is present in rat conjunctival goblet cells and can be activated by *S. aureus.* The NLRP3 inflammasome appears to contribute to inflammation in the conjunctiva by activating the secretion of IL-1β via the caspase 1 pathway. NLRP3 was previously reported to be highly expressed in wet mucosal epithelium within the cytoplasm of cells. [16] Kummer and colleagues [14] speculated that the expression of NLRP3 in such sites allows rapid sensing of invading pathogens or other danger signals, thereby triggering an innate immune response. The ocular surface is exposed to the environment, yet to maintain visual clarity, it is required to balance inflammation with immune privilege. Previous work demonstrated that mouse eyes express high levels of NLRP3 mRNA compared to other body tissues. [33] An additional study also reported NLRP3 mRNA expression in the whole eyes of mice challenged with LPS, but not in unchallenged eyes [34]. Benko et al previously reported NLRP3 mRNA was detected in human corneal epithelial cells, but was not detected at the protein level. [35] Our results show that NLRP3 protein, as well as the other constituent components of the NLRP3 inflammasome, are highly expressed in the goblet cells of the conjunctival epithelium.

In addition to the constitutive expression of NLRP3, we found that TLR2 is also expressed on the surface of rat goblet cells in culture, as were the purinergic receptors P2X4 and P2X7. Purinergic receptors are known to be activated by signals such as ATP, and TLRs by bacterial cell wall components, such as LTA. Both *S. aureus* and ATP are potent activators of the NLRP3 inflammasome, and we showed that ATP or LTA stimulation of goblet cells increases [Ca^2+^]_i_. Thus TLR2, P2X4, and P2X7 are indeed functional in goblet cell cultures, and could play a role in the priming and activation of the NLRP3 inflammasome. It was previously thought that caspase 1 regulation in macrophages required inflammatory stimuli that signalled through the TLRs to up-regulate gene products required for activation of the caspase 1 processing machinery and a second stimulus (such as ATP) to activate the inflammasome [36]. However, later work demonstrated that caspase 1 activation may be independent of TLRs. One study in particular demonstrated that pannexin-1 (a hemichannel protein that interacts with the P2X7 receptor) activation promotes cytosolic recognition of bacterial products to activate the NLRP3 inflammasome, which proceeds independent of TLR signalling. [37]. Our data demonstrate that ATP alone upregulates the expression NLRP3 in this inflammasome (Figures7) and activates caspase-1 (Figure 9), suggesting inflammasome activation. However, in the absence of a priming agent, there is a limited store of pro IL-1β to convert to mature IL-1β. Interestingly though, we demonstrate that goblet cells treated with *S. aureus* do not require ATP for inflammasome activation. In fact, *S. aureus* can act as both a priming agent, to upregulate pro IL-1β (Figure 8) and an activating agent to activate the inflammasome (Figure 9). The exact mechanism of NLRP3 inflammasome activation has not been fully elucidated in this study and requires further work with regards to TLR and P2X receptor involvement.

In this present study *S. aureus* challenge of rat goblet cells led to the activation of the NLRP3 inflammasome, demonstrated by the activation of caspase 1 and the secretion of IL-1β into the culture supernatants. We found that ATP, in combination with *S. aureus,* significantly enhanced IL-1β secretion compared to *S. aureus* alone, but this only reached significance in cultures treated with the higher MOI (MOI 60). This may be due to the amount of the pro form of IL-1β that is processed by the cell and made available for secretion, such that higher MOIs of *S. aureus* would result in more pro IL-1β being processed. Western blots revealed that pro IL-1β is present in rat goblet cells constitutively, which suggests that a small reserve is present and ready to become activated and released upon encounter with a danger trigger or pathogen. When the goblet cell cultures were stimulated with ATP alone, a small amount of IL-1β was secreted from the cultures into the supernatant, but this response failed to reach statistical significance when compared to untreated cells. Again this low response is most likely due to the limited amount of pro IL-1β available for processing and illustrates the importance of the priming agent. Bauernfeind et al [38] reported that NFκB activation was required for expression of pro IL-1β and NLRP3 protein and that the activation of the NLRP3 inflammasome was dependent on the level of NLRP3 expression. They found that the NLRP3 inflammasome was only activated in mouse macrophages that were first primed with a TLR agonist to activate the NFκB pathway, leading to the expression of NLRP3 and subsequent activation of the inflammasome via a NLRP3 agonist such as ATP. Our results provide evidence that although the inflammasome components are synthesized and pro IL-1β is constitutively present in the conjunctiva, IL-1β is not secreted until the activation step occurs. It is reported that in hematopoietic cells two signals are needed to activate the NLRP3 inflammasome and induce IL-1β secretion. These signals are: 1) a *TLR2 agonist* such as LTA from *S. aureus* to activate the NFκB pathway, which leads to enhanced expression of pro IL-1β and NLRP3; and 2) a *NLRP3 inflammasome agonist* such as a toxin (*e.g. S. aureus* alpha toxin, (the RN6390 strain used in this study produces alpha toxin)) or a danger signal (*e.g.* ATP). Our study shows that *S. aureus* increases the synthesis of pro IL-1β and also increases the secretion of IL-1β. Taken together, our data suggests that bacterial cell wall components, such as LTA, increases the expression of pro IL-1β while bacterial toxins, which have been previously shown to activate the inflammasome [6], [8], [10] may be responsible for the activation the NLRP3 inflammasome and secretion of active IL-1β in rat goblet cells.

The specific mechanism of NLRP3 inflammasome activation is currently under study. Several stimuli are known to activate the inflammasome and it is not clear as yet if all stimuli use the same method of activation. Tschopp and colleagues [1] were the first to coin the term inflammasome, and later showed that it could be activated by bacterial muramyl dipeptide. Further research has demonstrated the activation of the NLRP3 inflammasome in response to bacteria such as *S. aureus*. [6], [8]–[10] Recent literature suggests that TLR agonists such as LTA first activate transcription of IL-1β via the NFκB pathway; however processing of IL-1β to the active form appears to be initiated by *S. aureus* toxins, such as alpha toxin, which activates the inflammasome through an unknown mechanism. [6], [8], [10] Our study is consistent with these findings. We demonstrated that *S. aureus* leads to an increase in pro IL-1β protein expression and also to an increase in the expression of the NLRP3 inflammasome proteins. The *S. aureus* RN6390 strain used, which is known to produce toxins [26], also activated the caspase 1 pathway leading to IL-1β secretion from the cultures. Blocking of the caspase 1 pathway resulted in reduced secretion of IL-1β. Thus, in conjunctival goblet cells, *S. aureus* is sufficient to upregulate the expression of IL-1β and activate the NLRP3 inflammasome resulting in the secretion of active IL-1β.

Our data indicate that the NLRP3 inflammasome plays a role in initiating/promoting inflammation in the conjunctiva by activating IL-1β. The molecular mechanisms of how NLRP3 recognizes activators of the inflammasome in the conjunctiva remain to be elucidated, as does the sequence of events leading to ocular surface inflammation via the NLRP3 inflammasome. The inflammasome has been associated with specialized forms of cell death, pyronecrosis [39] (caspase 1 independent) and pyroptosis, [40] which may occur in cases of exacerbated inflammation. Ocular surface inflammation is associated with a reduced goblet cell density. [18], [19] We therefore hypothesize that normally the NLRP3 inflammasome is constitutively expressed for pathogen/danger surveillance, and activates an inflammatory response when triggered in order to protect the host tissue and eradicate the pathogen. However, the NLRP3 inflammasome may also play a pathophysiological role in chronic inflammatory states by inducing cell death when the acute inflammatory response fails. In the conjunctiva, subsequent goblet cell death may lead to lack of mucin on the ocular surface, which can enhance inflammation. [17].

We conclude that the components of the NLRP3 inflammasome are constitutively expressed in the conjunctival goblet cells. Moreover, exposure to a strain of *S. aureus* that is known to produce toxins associated with activation of the NLRP3 inflammasome, triggers the activation of caspase-1 and secretion of mature IL-1β, further supporting a *S. aureus-*mediated activation of the NLRP3 inflammasome in goblet cells. Additional studies with NLRP3 siRNA will confirm whether or not *S. aureus*-mediated activation of caspase-1 and secretion of mature IL-1β is solely dependent upon the NLRP3 inflammasome pathway. With further research into the exact mechanisms of activation, including clarification of the potential roles of the TLRs and P2X receptors, the NLRP3 inflammasome may prove to be a valuable target in development of new and more specific therapies for ocular surface inflammation.
